# The neural response to highly iconic signs in hearing learners and deaf signers

**DOI:** 10.1016/j.bandl.2025.105687

**Published:** 2025-12-12

**Authors:** Emily M. Akers, Katherine J. Midgley, Phillip J. Holcomb, Karen Emmorey

**Affiliations:** a Joint Doctoral Program in Language and Communicative Disorders, San Diego State University & University of California, 5500 Campanile Dr., San Diego, CA 92182, USA; b Department of Psychology, San Diego State University, 5500 Campanile Dr., San Diego, CA 92182, USA; c School of Speech, Language, and Hearing Sciences, San Diego State University, 5500 Campanile Dr., San Diego, CA 92182, USA

**Keywords:** Iconicity, American Sign Language, Event-related potentials, N400, Language learning

## Abstract

Previous ERP studies have demonstrated that hearing learners of American Sign Language (ASL) show sensitivity to sign iconicity (a resemblance between form and meaning) prior to learning any signs. Highly iconic (transparent) signs elicited greater negativity in the N400 window than non-iconic signs when participants performed a task that did not require semantic processing (detect an occasional grooming gesture). Greater negativity was interpreted as evidence that participants implicitly recognized the meaning of the iconic signs. Here we investigated how this neural response changes after learning. For comparison, we included a group of fluent deaf signers who performed the same task. Results revealed that the N400 to iconic signs became less negative after learning, indicating that these signs had been integrated into an emerging lexicon. In contrast, the N400 to non-iconic signs became more negative after learning, indicating more effortful processing compared to the iconic signs. For deaf signers, iconic signs elicited a larger N400 than non-iconic signs, which we interpret as a task effect whereby the highly iconic signs were seen as similar to the grooming gestures because both are enactments of actions (e.g., drinking from a cup; rubbing the eyes). In order to accurately perform the gesture detection task, deaf signers may have engaged in greater semantic processing of the iconic than non-iconic signs, which led to a larger N400 response. Overall, we conclude that iconicity modulates the neural response to signs in different ways before and after learning and that for deaf signers, iconicity effects are task dependent.

## 1. Introduction

Research on second language learning has most often focused on the acquisition of a second spoken language. In contrast, our research has examined how hearing speakers learn a new language in a different modality – a sign language. This brief report presents the third part of an ERP study that was conducted to investigate the effects of iconicity on sign language learning (Akers et al., 2024; Akers et al., 2025). Iconicity is characterized as the overlap between the linguistic form and the meaning of a sign or word. Since sign languages use the visual modality, there are more opportunities for signs to be iconic (e.g., it is easier to depict what an object looks like than what it sounds like) and for iconicity to interact with the learning and processing of signs.

Several previous studies have shown that iconic signs are easier for hearing adults to learn (Lieberth & Gamble, 1991; Morett, 2015; see Ortega, 2017, for review), which may be due to the overlap between iconic signs and common pantomimic gestures (because the structured mapping between the form and the meaning of an iconic sign may facilitate learning and memory (Emmorey, 2014). Specifically, iconicity may function like contextual support in word learning. For spoken language, novel words presented in semantically constraining contexts (*She piled pillows on the garn*) are learned better than those presented in unconstrained contexts (*Her favorite thing is garn*) (e.g., Abel et al., 2018). According to the Lexical Quality Hypothesis (Perfetti & Hart, 2002), a richer semantic context helps bind the form of a new word (its phonology) to its meaning. In addition, words learned within a richer semantic context exhibit a more robust N400 response than those learned in less supportive contexts (Frishkoff et al., 2010). Similarly, hearing learners show earlier and larger N400 priming effects for iconic than non-iconic signs (Mott et al., 2020), indicating that learned iconic signs have more stable and robust lexical representations.

Akers et al. (2025) did not examine sign learning, but rather used ERPs to investigate the initial neural response to iconic and non-iconic signs in two groups of hearing non-signers: those who were or were not expecting to learn American Sign Language (ASL) signs in the lab. The participants watched videos of non-iconic signs and highly iconic signs whose meaning was guessable from their form (e.g., the sign DRINK looks like the action of drinking from a cup). The participants’ task was to press a button whenever they saw an occasional grooming gesture (e.g., rubbing eyes). Note that this passive viewing of signs while pressing for gestures required no semantic processing of the stimuli. We found that when passively viewing signs, only participants expecting to later learn ASL signs showed an effect of iconicity: iconic signs produced greater negativity in the N400 window compared to non-iconic signs. Because the N400 has been consistently related to meaning-based processing (Kutas & Hillyard, 1980; Kutas & Federmeier, 2011), we interpreted greater negativity for highly iconic signs as indicating greater semantic processing, and we suggested that participants in the “learner” group attempted to attach meaning to signs before they even began to learn them. The fact that participants in the “non-learner” group did not show this effect indicates that hearing non-signers do not automatically process the meaning of highly iconic signs, unless there is a motivational factor (i.e., the expectation to learn signs). Here, we investigated whether and how the neural response to iconic and non-iconic signs observed in the “learner” group changes after they learn the meaning of the signs. That is, does the neural sensitivity to iconicity during the passive viewing of signs change after learning, and if so, how?

We do know how the neural response to these highly iconic and non-iconic signs changes with learning when the task requires an explicit meaning judgment. Specifically, the same participants from the learner group in Akers et al. (2025) also performed a word-sign matching task before and after learning – these results were reported in Akers et al. (2024). In this task (which was performed after the gesture detection task), participants saw a word followed by an ASL sign and were asked to decide (or guess, pre-learning) if the word was the correct translation of the sign. Learning occurred in the lab over the course of a few days by associating each ASL sign with its English translation. Before learning, highly iconic signs exhibited a typical priming effect, i.e., larger N400 for unrelated trials (mismatched word-sign translations) than for related trials (correct word-sign translations), while the non-iconic signs did not show a difference in N400 amplitude. This finding again indicates that participants had already begun to assign meaning to these easily interpretable signs and is consistent with the results of Akers et al. (2025) described above. After learning the signs, non-iconic signs exhibited an N400 priming effect (greater negativity for unrelated than related trials), but this priming effect decreased after learning for iconic signs. The reduction in N400 priming for the iconic signs indicates that the learners had consolidated stable form-meaning mappings for these signs. For deaf ASL signers, iconicity did not modulate the size of the N400 priming effect. Akers et al. (2024) concluded that the impact of iconicity on lexico-semantic processing is reduced following learning.

In the current study, we examined how neural sensitivity to iconicity changes with learning when the task does not require semantic processing, i.e., the grooming gesture detection task. Previously, Ortega et al. (2020) used passive viewing without an explicit task and found that before learning, iconic signs whose form did not overlap with pantomimic gesture elicited a larger P300 (an index of expectation) compared to iconic signs with high gesture overlap, and this effect disappeared after learning. Because all signs were iconic, participants in this study were likely generating expectations about the form of the signs, eliciting a P300 response. In contrast, here we compare the pre-learning N400 results for highly iconic and non-iconic signs reported by Akers et al. (2025) for the learner group to their post-learning ERP data (not reported in Akers et al., 2025). Our pre-registered hypothesis was that the hearing learners would continue to show greater negativity for highly iconic signs compared to non-iconic signs, but that this difference would be reduced after learning. This pattern would indicate that both sign types had been integrated into the lexicon, but that iconic signs continue to elicit more semantic activation (akin to concrete words; cf. McGarry et al., 2021). However, another possibility is that non-iconic signs show greater negativity than iconic signs after learning. This result would indicate that non-iconic signs have not yet been consolidated into stable lexical representations and are less well learned than highly iconic signs (akin to a larger N400 for less familiar words). A third possibility is that iconic and non-iconic signs do not differ from each other after learning, indicating that both sign types have been equally integrated into the lexicon.

In addition, we examined the neural response for the deaf signers from Akers et al. (2024) when they passively viewed the iconic and non-iconic signs and performed the gesture detection task (previously unreported results). We did not anticipate an effect of iconicity given that for deaf signers iconicity does not modulate the size of the priming effect in word-sign matching tasks (Akers et al., 2024; Mott et al., 2020). However, iconicity effects have proven to be task-dependent for fluent deaf signers. For example, iconicity has been found to facilitate sign production in picture-naming tasks (e.g., Baus & Costa, 2015; McGarry et al., 2021; Navarrete et al., 2017), to slow phonological decisions about sign form (Thompson et al., 2010), and to have no effect on lexical decision (Bosworth & Emmorey, 2010) or translation tasks (Gimeno-Martinez & Baus, 2022; McGarry et al., 2023). Given this variability, it is possible that some aspects of the gesture detection task could be influenced by sign iconicity for deaf signers. Overall, the current study adds to the existing literature by a) assessing the possible neural effect of iconicity on sign recognition when deaf signers perform a gesture-related task and b) providing new insight into the neural mechanisms associated with word learning by identifying how the N400 response elicited during passive viewing changes after learning for highly iconic vs. non-iconic signs.

## 2. Method

This project was preregistered. Preregistration information (https://osf.io/r3h6d), as well as data and materials (https://osf.io/zq5ax/), are available on the project’s Open Science Framework (OSF) page. Below is a brief summary of the methods from Akers et al. (2024). For the full, detailed methods section, please see the Supplementary Materials.

The hearing sign-naïve participants (n = 32) from Akers et al. (2024) had been recruited to participate in a 3-day sign language learning study. They were taught 100 ASL signs (half highly iconic and half non-iconic) using a word-sign association paradigm, and ERPs were collected before and after learning. As a comparison group, 20 deaf native/early signers also participated in the ERP session (without the learning sessions). All participants performed a gesture detection task where they responded on a button box when they saw the signer perform an occasional grooming gesture (e.g., scratching eyes). Following Akers et al. (2024) and our pre-registered analysis plan, we examined four time windows: 400–600 ms, 600–800 ms, 800–1000 ms, and 1000–1400 ms, and we analyzed nine central electrode sites (F3, Fz, F4, C3, Cz, C4, P3, Pz, P4). This subset of electrode sites was selected based on our previous learning studies (Mott et al., 2020; Pu et al., 2016) and to give adequate scalp coverage while allowing for a single ANOVA (including scalp distribution) per epoch.

## 3. Results

### 3.1. Behavioral results: gesture detection task

#### 3.1.1. Hearing learners

There was a significant main effect of Learning (F(1,31) = 80.67, *p* < 0.0001), indicating that there were fewer false alarms after learning (0.36% of trials) than before learning (11.5% of trials) and a main effect of Iconicity (F(1,31) = 20.01, *p* = 0.0001), indicating that iconic signs (before learning 18.2%; after learning 0.50%) produced more false alarms than non-iconic signs (before learning 4.9%; after learning 0.44%). There was also a significant interaction between Learning and Iconicity (F(1,31) = 20.39, *p* = 0.0001), showing that the difference in false alarms between iconic and non-iconic signs was reduced after learning.

#### 3.1.2. Deaf signers

There was a significant main effect of Iconicity (F(1,19) = 13.14, *p* = 0.0018) – iconic signs (16.7% of trials) produced more false alarms than non-iconic signs (4.2% of trials).

### 3.2. ERP results

#### 3.2.1. Hearing learners: before and after learning

**Fig. 1.**
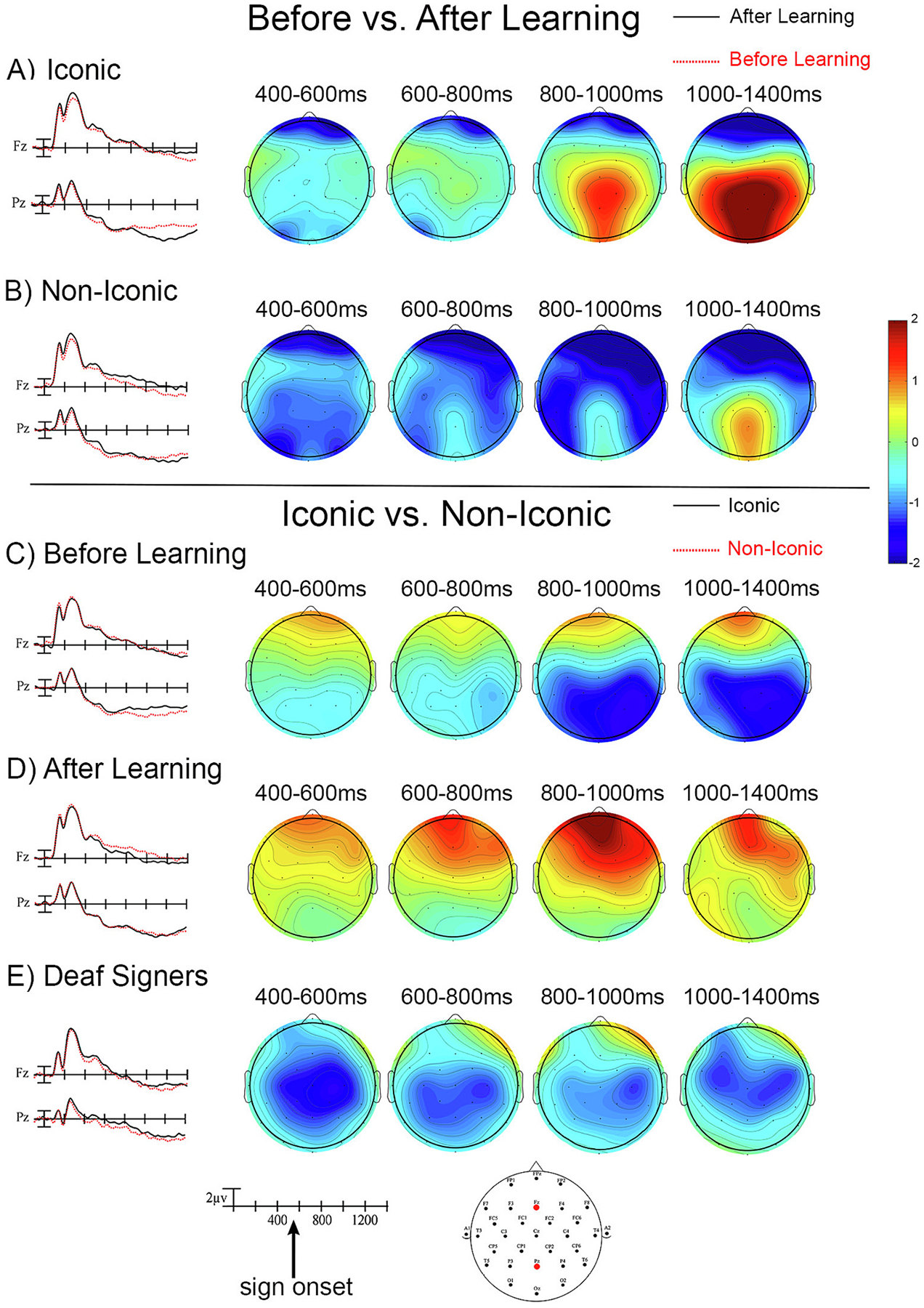
ERP waves at sites Fz (fronto-central) and Pz (central-parietal) and voltage maps for all four epochs. The Fz and Pz electrodes are in red on the montage, and sign onset is indicated with an arrow at ~578 ms. For panels A and B voltage maps were created by subtracting after learning trials (black ERP line) from before learning trials (red ERP line) – blue color indicates greater negativity after learning, while red indicates greater negativity before learning. For panels C-E, voltage maps were created by subtracting ERPs to non-iconic signs from iconic signs in the four epochs of interest – blue color indicates greater negativity for iconic signs, whereas red indicates greater negativity for non-iconic signs. Panels A and B illustrate the ERP waves and voltage maps for iconic signs (A) and non-iconic signs (B) in the hearing learners before vs. after learning. Panels C and D illustrate ERP waves and voltage maps for iconic vs. non-iconic signs in hearing learners before learning (C) and after learning (D). Panel E illustrates ERP waves and voltage maps for iconic vs. non-iconic signs for deaf signers. All waves are plotted negative up.

Fig. 1 shows the ERPs recorded to target signs in the gesture detection task, as well as voltage maps for all four time epochs. Fig. 1A and 1B illustrate changes with learning for iconic and non-iconic signs, respectively, while Fig. 1C and 1D illustrate the response to both iconic and non-iconic signs for hearing learners before and after learning, respectively. Fig. 1E illustrates the response to iconic and non-iconic signs for the deaf signers. We considered the 800–100 ms epoch to be the N400 time window for hearing learners because it is about 300–500 ms post-sign-onset – mean sign onset time was 578 ms. Sign onset was defined following Caselli et al. (2017) – briefly, sign onset is the first video frame when the hand contacts the body or when it reaches a target location in neutral space. ERPs were time-locked to video onset and averaged across the nine central sites for each of the four time windows.

##### 400–600 ms time epoch.

There were no significant differences between Learning and Iconicity for this time window (all *p* > 0.10).

##### 600–800 ms time epoch.

There were no significant differences between Learning and Iconicity for this time window (all *p* > 0.15).

##### 800–1000 ms time epoch (N400 window).

For this time window, there was a significant interaction between Learning and Iconicity (F (1,31) = 8.88, *p* = 0.0056, $\eta_{\text{p}}^{2}$ = 0.2226), suggesting that before learning, iconic signs were more negative-going than non-iconic signs, but after learning, the pattern reversed with non-iconic signs showing more negative-going waves. To verify this observation, we conducted follow-up analyses examining iconic and non-iconic signs separately. For iconic signs, there was a significant interaction between Learning, Laterality, and Anteriority (F(4,124) = 3.2, *p* = 0.0366, $\eta_{\text{p}}^{2}$ = 0.0937) such that iconic signs became less negative with learning, especially at central posterior sites (see Fig. 1A). For non-iconic signs, there was a main effect of Learning (F(1,31) = 5.72, *p* = 0.023, $\eta_{\text{p}}^{2}$ = 0.1558) as well as a significant interaction between Learning, Laterality, and Anteriority (F (4,124) = 7.02, *p* = 0.0004, $\eta_{\text{p}}^{2}$ = 0.1846) indicating that non-iconic signs became more negative with learning especially at central anterior sites (see Fig. 1B).

##### 1000–1400 ms time epoch.

For this fourth time window, there was a significant interaction between Learning and Iconicity (F(1,31) = 5.82, *p* = 0.022, $\eta_{\text{p}}^{2}$ = 0.158) – the pattern from the previous window continued into this epoch: before learning iconic signs showed more negativity than non-iconic signs, but after learning this pattern flipped with non-iconic signs showing more negativity than iconic signs. We again conducted follow-up analyses comparing iconic signs and non-iconic signs separately to further investigate the interaction between Learning and Iconicity. For iconic signs, the significant interaction between Learning, Laterality, and Anteriority continued (F(4,124) = 5.06, *p* = 0.0065, $\eta_{\text{p}}^{2}$ = 0.1403) with iconic signs eliciting less negativity after learning, especially over more central posterior sites (see Fig. 1A). For non-iconic signs, the previous epoch pattern also continued with a significant interaction between Learning, Laterality, and Anteriority (F (4,124) = 6.44, *p* = 0.0012, $\eta_{\text{p}}^{2}$ = 0.172). After learning non-iconic signs produced greater central anterior negativity (see Fig. 1B).

#### 3.2.2. Deaf signers

Previous research indicates that deaf fluent signers use transitional information to begin lexical access before sign onset (Emmorey et al., 2022; Mott et al., 2020), and therefore we considered an earlier epoch (600–800 ms) to be the N400 time window for the deaf signer group.

##### 400–600 ms time epoch.

For this first time window, there was a main effect for Iconicity (F(1,19) = 9.1, *p* = 0.0071, $\eta_{\text{p}}^{2}$ = 0.3237) – iconic signs elicited greater negativity than non-iconic signs (Fig. 1E).

##### 600–800 ms time epoch (N400 window).

For this time window, there was a significant interaction between Iconicity and Anteriority (F (2,38) = 3.85, *p* = 0.0394, $\eta_{\text{p}}^{2}$ = 0.1686) with greater negative-going ERPs for iconic than non-iconic signs, especially over centro-parietal sites.

##### 800–1000 ms time epoch.

There were no significant effects for this time window (all p > 0.12).

##### 1000–1400 ms time epoch.

For this fourth time window, there was again a significant main effect of Iconicity (F(1,19) = 5.04, p = 0.0369, $\eta_{\text{p}}^{2}$ = 0.2097) with greater negativity for iconic signs.

## 4. Discussion

The results revealed that after learning, non-iconic signs elicited a larger N400 (more negative response) than highly iconic signs (see Fig. 1D), which is contrary to our pre-registered hypothesis that iconic signs would continue to elicit a larger N400 than non-iconic signs because iconic signs activate more semantic features (akin to concrete words). However, this finding is consistent with the alternative hypothesis that learned non-iconic signs had less stable lexical representations and were less recognizable than highly iconic signs, resulting in a larger N400 response. In fact, prior to learning, the meanings of the highly iconic signs were easily guessed by the participants. Accuracy on the word-sign matching task for iconic signs was not significantly different for the pre- vs. post-learning sessions (96% vs.99 %), whereas non-iconic signs had a much steeper learning curve (64% vs. 98%) (Akers et al., 2024). The larger amplitude N400 for non-iconic signs indicates that more neural activity was required to access their meaning compared to the iconic signs.

However, the distribution of the increased negativity for non-iconic signs after learning was more anterior than the typical centro-posterior N400 response. Previous work in second language learners, especially those at the early stages of L2 acquisition, have noted similar anteriorly distributed negativities (e.g., Midgley et al., 2009; Pu et al., 2016; Yum et al., 2014). Midgley et al. (2009) suggested that the anterior distribution is consistent with a process whereby new L2 words are translated into L1 prior to semantic activation as predicted by bilingual processing models such as the Revised Hierarchical Model (Kroll & Stewart, 1994). This interpretation would seem to fit with the effect observed here with new learners of ASL signs showing a larger anterior negativity after learning. This interpretation also fits with the larger central-anterior negativities after learning for the non-iconic than iconic signs since learners most likely would need to translate the non-iconic signs, but not the highly iconic signs because such signs should activate semantic representations without the need for translation.

As noted above, we did not find that iconic signs continued to show greater negativity compared to non-iconic signs after learning (see Fig. 1A compared to Fig. 1B). In fact, the negativity to iconic signs decreased after learning (*p* = 0.0366; see Fig. 1A). This finding is consistent with the reduction in the N400 priming effect for iconic signs that occurred after learning in the word-sign matching task (Akers et al., 2024). Both of these findings indicate that hearing learners already had established semantic representations for these highly iconic signs (perhaps based on their gestural knowledge) prior to learning. After the learning sessions, the neural response to these iconic signs became less negative because they were now more familiar and more frequent. Many previous ERP studies have shown that more frequent items have a smaller N400 compared to less frequent items (Dufau et al., 2015; Emmorey et al., 2020).

Somewhat surprisingly, the deaf signers also showed an iconicity effect (see Fig. 1E). These results were unexpected because these same deaf signers saw the same signs in a word-sign matching task, where sign iconicity did not modulate the size of the priming effect (Akers et al., 2024). However, we suspect that differences in task demands between the grooming gesture detection task and the word-sign matching task may explain this finding. Specifically, for the word-sign matching task, there was no relationship between the English word and the iconicity of the ASL signs, and thus iconicity did not modulate the priming effect (i.e., larger N400 for unrelated than translation-matched pairs), as also found by Mott et al. (2020). However, the grooming gesture detection task may have influenced how the highly iconic signs used in this study were recognized. That is, these iconic signs were strongly enactive and depictive of the actions they denoted (e.g., drinking from a cup; brushing hair). Although the grooming gestures were not ASL signs, they were enactments and could be produced in a narrative or conversation as constructed actions (Cormier et al., 2015). For example, the gesture “rubbing eyes” could be used to depict the process of someone waking up in an ASL story. Given the depictive and enactment parallels between the grooming gestures and the highly iconic signs, we speculate that it was more difficult for the deaf signers to distinguish between gestures and iconic signs. Some suggestive evidence for this hypothesis comes from the behavioral data. Deaf signers made more false alarms to iconic than non-iconic signs, as did the hearing learners. This result suggests that these highly iconic signs were seen as depictive enactments for both groups of participants. For the deaf signers, we suggest that this overlap between the target grooming gestures and the highly iconic signs lead to increased lexical processing for iconic signs compared to non-iconic signs in order to accurately perform the gesture detection task. Such increased lexical processing would lead to greater negativity for the iconic than non-iconic signs.

In summary, learners exhibited a flip in polarity after learning, such that before learning, iconic signs elicited a more negative response, but after learning, non-iconic signs elicited a more negative response. We suggest that after learning the non-iconic signs had less stable lexical representations, were more difficult to recognize, and were more likely to be translated into English before semantic activation. In contrast, the meanings of the iconic signs were recognizable from the beginning, and thus the reduction in N400 amplitude after learning may reflect the establishment of more stable lexical representations, easier recognition, and less need to translate to English to engage semantic processing. However, it is important to note that the learning that took place for this study was short-term and in a lab setting. Future research could examine how long-term and more natural ASL learning might impact the passive processing of highly iconic and non-iconic signs. For deaf signers, iconic signs elicited a larger N400 than non-iconic signs, which we attribute to the demands of the gesture detection task (i.e., the need to discriminate between different types of enactments). We conclude that iconicity modulates the neural response for sign language learners both before and after learning and that for fluent deaf signers, the neural effects of iconicity are task dependent.

## Supplementary Material

Supplementary Material

## Data Availability

All data and materials have been uploaded to OSF and can be accessed at the following link: https://osf.io/zq5ax/.

## Appendix A. Supplementary data

Supplementary data to this article can be found online at https://doi.org/10.1016/j.bandl.2025.105687.
