# Supplementary material for: The neural response to highly iconic signs in hearing learners and deaf signers
Source: Brain Lang. Author manuscript; Available in PMC 2026 Feb 1. (PMC12831478; doi:10.1016/j.bandl.2025.105687)
Supplement: Supplementary Material [file NIHMS2139399-supplement-Supplementary_Material.docx]

The methods reported below are compiled from the following previously published studies:

Akers, E., Midgley, K.J., Holcomb, P.J., Meade, G., & Emmorey, K. (2024). Neural effects differ for learning highly iconic versus non-iconic signs in hearing adults. *Bilingualism: Language and Cognition*, 27(4), 655-667. doi:10.1017/S1366728923000809

Akers, E.M., Midgley, K.J., Holcomb, P.J., & Emmorey, K. (2025). Semantic processing of iconic signs is not automatic: Neural evidence from hearing non-signers. *Bilingualism: Language and Cognition*. <https://doi.org/10.1017/S1366728924001093>

**Methods**

This project was preregistered. Preregistration information (<https://osf.io/r3h6d>), as well as data and materials (<https://osf.io/zq5ax/>), are available at the project’s Open Science Framework (OSF) page.

**Participants**

Participants in the learning group were 32 hearing English-speaking adults with no prior exposure to ASL (18 females; mean age 21 years, SD = 2.37, range = 18 – 29 years). All participants were right-handed and had either normal or corrected-to-normal vision. Participants reported no history of neurological disorders or learning impairments. They were all recruited from San Diego State University and the surrounding area.

Participants in the signer group were 20 deaf American Sign Language (ASL) signers, all of whom became deaf before or at two years old (8 females; mean age 34 years, SD = 10.04, range = 20 – 54 years). Twelve participants were native signers (born into signing families), eight participants were early signers (acquired ASL prior to age six), and all reported ASL as their preferred language. Seventeen participants were right-handed, one was one left-handed, and two reported being ambidextrous. Two participants had a cochlear implant, and the external sound processor was removed before collecting data. All deaf participants had either normal or corrected-to-normal vision and reported no history of neurological disorders or learning impairments.

All participants were treated in accordance with SDSU IRB guidelines. They provided informed consent and were given monetary compensation for their participation.

**Stimuli**

The stimuli consisted of 100 video clips of ASL signs produced by a native female signer. Videos were presented on an LCD video monitor while the participants sat 110cm (43in) away from the screen. The video size was 10 x 13.25cm in the center of the screen with a visual angle of 5.21 x 6.89 degrees. The signer was positioned in the middle of the frame so that her signing could be perceived without the participant needing to move their eyes. Note that even novice ASL learners look at the signer’s face and do not track the hands (Emmorey, Thompson, & Colvin, 2009). All videos started with the sign model in a resting position with her hands on her lap and ended when her hands returned to her lap. The average video length was 2157ms (SD = 290ms), with an average sign onset of 578ms (SD = 104ms). Sign onset was determined as in Caselli et al. (2017). Briefly, sign onset is the first video frame in which the fully formed handshape contacts the body, and if the sign does not have body contact, then onset is defined as the first video frame in which the fully formed handshape arrives at the target location near the body or in neutral space before starting the sign movement. The average grooming gesture video length was 3145ms (SD = 379ms), with an average gesture onset of 545ms (SD = 114ms). Examples of grooming gestures included the sign model rubbing her eyes, picking her fingernails, scratching her head, and adjusting her clothing.

Fifty highly iconic signs were selected based on ratings from the ASL-LEX database (http://asl-lex.org; Caselli et al., 2017; Sehyr et al., 2021). Iconicity ratings were completed by hearing non-signers using a scale of 1 (not iconic) to 7 (very iconic). The iconic signs all had ratings over 5.0 (M = 6.3, SD = .51). In addition, to help ensure that the meanings of iconic signs were relatively transparent or “guessable”, we utilized the transparency ratings from the ASL-LEX database and collected additional ratings when transparency information was not available in the database. To gather transparency ratings, American hearing non-signers were asked to guess the meaning of an ASL sign and then to rate how obvious their guessed meaning would be to others, on a scale of 1 (not obvious at all) to 7 (very obvious). All iconic signs had a transparency rating of over 4.0 (M = 5.05, SD = .60). Examples of highly iconic, transparent signs are [CIRCLE](https://asl-lex.org/visualization/?sign=circle) (index finger traces a circle in the air) and [BRUSH](https://asl-lex.org/visualization/?sign=brush) (depicts brushing one’s hair); video links for all signs and gestures are on the project’s OSF page in the stimuli document (<https://osf.io/zq5ax/>). The average video length for the iconic signs was 2189ms (SD = 331ms), and the average sign onset within the video was 569ms (SD = 97ms).

The other 50 signs^^[[1]](#footnote-1)^^ were non-iconic with an average video length of 2124ms (SD = 242ms), and an average sign onset within the video of 587ms (SD = 111ms). These signs had iconicity ratings under 3.0 (M = 1.92, SD = .47) and transparency ratings under 4.0 (M = 3.37, SD = .34). The iconic and non-iconic signs were also matched on ASL sign frequency (based on ratings from ASL-LEX), word frequency of their English translations (Vanheuven et al., 2014), concreteness of their English translations (Brysbaert et al., 2014), number of hands used in the sign, length of video, and average sign onset. SM Table 1 provides descriptive statistics for the sign stimuli.

|  | Concreteness M (SD) | Word Frequency M (SD) | Sign Frequency M (SD) | Iconicity  M (SD) | Transparency M (SD) | Duration  M (SD) | Sign Onset  M (SD) |
| --- | --- | --- | --- | --- | --- | --- | --- |
| Iconic | 3.96 (.84) | 4.52 (.74) | 4.16 (1.16) | 6.31 (.51) | 5.05 (.60) | 2189ms (331ms) | 569ms (97ms) |
| Non- Iconic | 3.82 (.89) | 4.40 (.65) | 4.15 (1.14) | 1.92 (.47) | 3.37 (.34) | 2124ms (242ms) | 587ms (111ms) |
|  | *p* = .421 | *p* = .386 | *p* = .990 | *p* < .001 | *p* < .001 | *p* = .270 | *p* = .406 |

**SM Table 1.** Means and standard deviations for the descriptive characteristics of the iconic and non-iconic signs. *p*-values reported beneath each comparison reflect the t-test results for the relevant comparison between the iconic and non-iconic signs.

**Procedure**

**ERP Sessions**

The ERP session consisted of a gesture detection task in which participants passively viewed the signs and pressed a button on a gamepad when they detected a grooming gesture. Before learning, learners were told that they would see videos of signs and their task was to identify a video that looked like a gesture and not sign language, such as when the signer scratched her head or stretched out her arms (demonstrated by the experimenter). After learning and for the deaf participants, they were told to press when they saw a grooming gesture and not a sign.

Each trial began with a white fixation cross for 500ms followed by a blank screen for 500ms. Immediately after the blank screen a grooming gesture or a sign video was presented. After this, a trial-ending 800ms purple fixation was displayed indicating it was OK to blink before the beginning of the next trial. Participants were asked to respond as quickly and as accurately as they could (see SM Figure 1A for a schematic of a typical trial). All other stimuli (i.e., the ASL signs) did not require a button press.


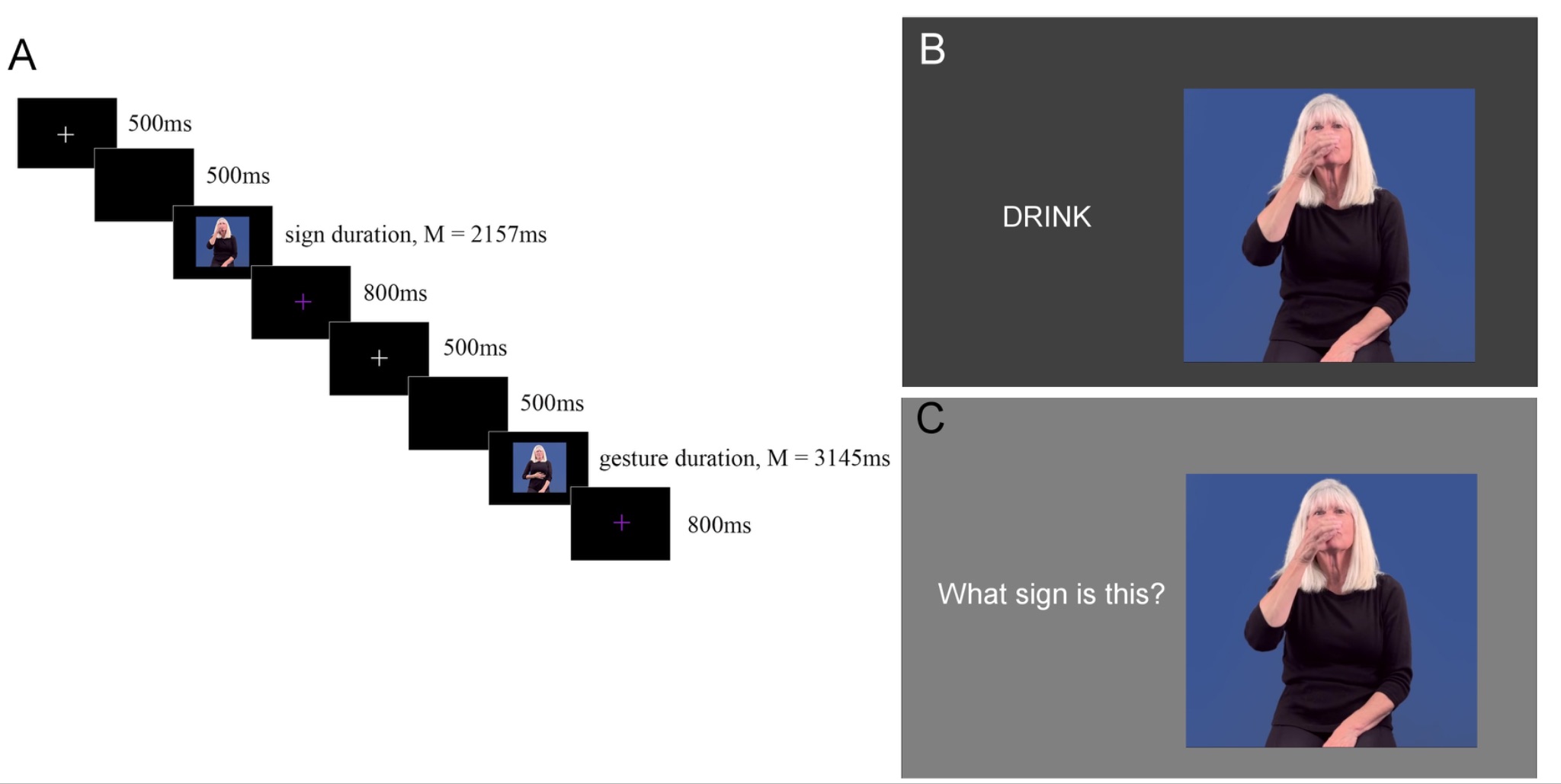


**SM Figure 1.** **A)** Schematic of a typical trial in the grooming gesture detection ERP session. **B)** Typical trial in the learning task. The English translation was presented simultaneously with a video of the ASL sign. **C)** Typical trial in the test section of learning task. Participants gave their answer verbally to the experimenter in the room.

There were two stimulus lists, which contained the same signs and gestures but in reverse presentation order. The lists were counterbalanced across participants. Both lists were pseudo-randomized so that no more than three trials in a row were in the same condition (iconic or non-iconic). Six additional signs (3 iconic and 3 non-iconic) and two grooming gestures were used in a short practice session prior to the ERP session to introduce the task to the participants and to provide time for any questions. These trials were not included in the analyses but were learned during the learning sessions for the hearing learners.

**Learning Sessions**

Hearing participants received two learning sessions and four testing sessions. After the first ERP session in which participants performed both the gesture detection task performed in the current study and the English word sign matching task performed in Akers et al. (2024), participants were brought into a new room to learn all the signs they had just seen. They were shown a PowerPoint slideshow where a video of each sign was presented next to its English translation (see SM Figure 1B for a typical learning trial). Participants were able to replay the videos of the signs, and they were told to try to remember each sign using whatever strategy they wished.

After participants had watched all the signs with their English translations, they were tested. Participants watched another PowerPoint with the signs in a different order and without the English words. For each sign, they were instructed to tell a researcher in the room what they thought the English translation was (see SM Figure 1C for a typical test trial). The researcher gave participants feedback and, if wrong, told them the correct English translation. This testing session was completed on the first day of the study (after the ERP recording session).

In the second learning session (one or two days after the first ERP/learning session), participants met with the researcher via Zoom. They were tested again to see how much they remembered from the previous session using the same testing format with the researcher’s screen shared with the participant. After this second testing session, participants undertook another learning session where they saw all the signs again with their English translations. Then they were tested for a third time.

On the third session (one to two days after the second learning session), after completing the ERP session (the gesture detection task and word-sign matching task), participants were tested one last time to assess how well they remembered all the signs.

For each learning and testing session, the order of signs in the PowerPoint presentation was pseudo-randomized, such that no more than four iconic or non-iconic signs were in a consecutive order. Participants were told not to practice any of the signs outside of the experiment sessions during the week of testing.

**EEG Recording**

All participants were seated in a comfortable chair in a darkened, sound attenuating room. EEG was continuously recorded through a 29-channel cap with tin electrodes (Electro-cap International, Inc., Eaton, OH). There were four loose electrodes placed on the participant’s head at the following locations: one underneath the left eye to track blinking, one on the side of the right eye to track horizontal eye movements, and one placed on each mastoid bone behind the ear- the left mastoid was used as the reference electrode. All electrodes were connected using a saline-based gel (Electro-Gel), and impedances were reduced to under 2.5kΩ. The data was collected through Curry Data Acquisition software with a sampling rate of 500Hz, and the EEG signal was amplified by a SynAmpsRT amplifier (Neuroscan-Compumedics, Charlotte, NC) with a bandpass of DC to 100Hz.

ERPs were time-locked offline to the onset of the target ASL sign (video onset) with a 100ms pre-stimulus baseline. To remove eye blinks and other eye artifacts prior to data analysis, Independent Component Analysis (ICA) from the EEGLAB function under MATLAB was used (Makeig et al., 1996). These components were removed from the data prior to averaging (between one and three components were removed per participant). ERPs from individual sites were processed with a 15 Hz low-pass filter prior to analysis. Trials that had artifact post-ICA were removed from the analysis (post-ICA: before learning = 0.47% trials rejected; after learning = 1% trials rejected; deaf signers = 0.1%).

**Data Analysis**

False alarms and accuracy were measured for the gesture detection task that was performed while ERPs were recorded before and after learning.

For the analysis of the ERP data, following Mott et al. (2020) and Akers et al. (2025), ERPs were time-locked to video onset, and nine electrode sites were analyzed to identify effects across a representative sample of scalp sites (see SM Figure 2 for the sites analyzed). Prior language learning studies in our lab have shown that this grid analysis approach offers the best coverage of the scalp with the fewest number of statistical comparisons (e.g., Yum et al., 2014). Also following Mott et al. (2020), Akers et al. (2024), and Akers et al. (2025), we quantified the ERP data in four windows: 400–600ms, 600-800ms, 800–1000ms, and 1000–1400ms (baselined to the mean amplitude in the 100ms pre-stimulus epoch). Since we time-locked to video onset, and the average sign onset occurred at 578ms, we expected N400 effects to be most notable in the 800-1000ms time-window.

For the learning group, a within-subjects ANOVA was used to assess differences as a function of Learning (before learning vs. after learning), Iconicity (iconic vs. non-iconic), and any interactions with scalp distribution. Scalp distribution had two factors; Anteriority (frontal, central, and occipital) and Laterality (left, middle, right). For effects that showed a learning difference, separate analyses were performed to examine the effects of iconic and non-iconic signs separately and how they interacted with the scalp distributions (anteriority and laterality). For the deaf signers, a similar within-subjects ANOVA was performed to assess how Iconicity interacted with the two scalp distributions.

Significant results (p < .05) are reported in the manuscript. Partial eta squared (η_p_^2^) is reported as a measure of effect size, and Greenhouse-Geisser (1959) correction was used for all significant effects with a degree of freedom numerator greater than one.


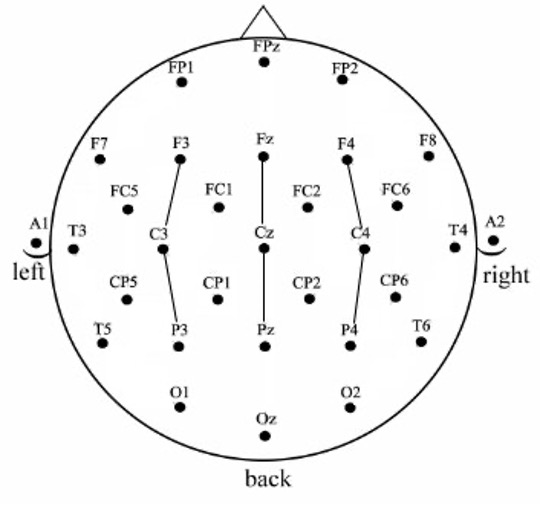


**SM Figure 2**. Electrode montage and nine analysis sites (sites connected with lines)

**References**

Akers, E. M., Midgley, K. J., Holcomb, P. J., & Emmorey, K. (2025). Semantic processing of iconic signs is not automatic: Neural evidence from hearing non-signers. *Bilingualism: Language and Cognition*, 1–9. <https://doi.org/10.1017/S1366728924001093>

Akers, E. M., Midgley, K. J., Holcomb, P. J., Meade, G., & Emmorey, K. (2024). Neural effects differ for learning highly iconic versus non-iconic signs in hearing adults. *Bilingualism: Language and Cognition*, 1–13. <https://doi.org/10.1017/S1366728923000809>

Brysbaert, M., Warriner, A.B., & Kuperman, V. (2014). Concreteness ratings for 40 thousand generally known English word lemmas. *Behavior Research Methods*, 46, 904-911.Campbell, R., Martin, P., & White, T. (1992). Forced choice recognition of sign in novice learners of British Sign Language. *Applied Linguistics*, *13*(2), 185–201. <https://doi.org/10.1093/applin/13.2.185>

Caselli, N. K., Sehyr, Z. S., Cohen-Goldberg, A. M., & Emmorey, K. (2017). ASL-LEX: A lexical database of American Sign Language. *Behavior Research Methods*, 49(2), 784–801. <https://doi.org/10.3758/s13428-016-0742-0>

Emmorey, K., Thompson, R., & Colvin, R. (2009). Eye gaze during comprehension of American Sign Language by native and beginning signers. *Journal of Deaf Studies and Deaf Education*, 14(2), 237-43. <https://doi.org/10.1093/deafed/enn037>

Greenhouse, S. W., & Geisser, S. (1959). On methods in the analysis of profile data. *Psychometrika*, 24(2), 95–112. <https://doi.org/10.1007/BF02289823>

Makeig, S., Bell, A. J., Jung, T. P., & Sejnowski, T. J. (1996). Independent component analysis of electroencephalographic data. *In Advances in neural information processing systems* (pp. 145-151).

Mott, M., Midgley, K.J., Holcomb, P.J., Emmorey, K. (2020) Cross-modal translation priming and iconicity effects in deaf signers and hearing learners of American Sign Language, *Bilingualism: Language and Cognition*. <https://doi.org/10.1017/S1366728919000889>

Sehyr, Z.S., Caselli, N., Cohen-Goldberg, A., Emmorey, K. (2021). The ASL-LEX 2.0 Project: A database of lexical and phonological properties for 2,723 signs in American Sign Language. *Journal of Deaf Studies and Deaf Education*, 26(2), 263-277. <https://doi.org/10.1093/deafed/enaa038>

Van Heuven, W. J., Mandera, P., Keuleers, E., & Brysbaert, M. (2014). SUBTLEX-UK: A new and improved word frequency database for British English. *Quarterly Journal of Experimental Psychology*, 67(6), 1176–1190. <http://dx.doi.org/10.1080/17470218.2013.850521>

Yum, Y. N., Midgley, K. J., Holcomb, P. J., & Grainger, J. (2014). An ERP study on initial second language vocabulary learning: Initial L2 vocabulary learning. *Psychophysiology*, *51*(4), 364–373. <https://doi.org/10.1111/psyp.12183>

1. For the deaf participants there were 47 non-iconic signs because three were removed, one had two possible English translations (“Atlanta” and “assembly”), one was very low frequency ([SPACESHIP](https://asl-lex.org/visualization/?sign=spaceship)), and one had regional variations ([KANGAROO](https://asl-lex.org/visualization/?sign=kangaroo_3)). [↑](#footnote-ref-1)
